# Intra-respiratory variation in tumour–diaphragm geometric association as a potential surrogate for dynamic tumour-tracking radiotherapy

**DOI:** 10.1016/j.phro.2026.101057

**Published:** 2026-08-04

**Authors:** Takahiro Iwai, Masahiro Yoneyama, Noriko Kishi, Takanori Adachi, Mitsuhiro Nakamura, Yukine Shimizu, Hideaki Hirashima, Nobutaka Mukumoto, Michio Yoshimura, Takashi Mizowaki

**Affiliations:** a Department of Radiation Oncology and Image-Applied Therapy, Graduate School of Medicine, Kyoto University, 54 Kawahara-cho, Shogoin, Sakyo-ku, Kyoto, 606-8507, Japan.; b Department of Advanced Medical Physics, Graduate School of Medicine, Kyoto University, 53 Kawahara-cho, Shogoin, Sakyo-ku, Kyoto, 606-8507, Japan.; c Department of Radiation Oncology, Graduate School of Medicine, Osaka Metropolitan University, 1-4-3 Asahi-machi, Abeno-ku, Osaka, 545-0051, Japan.

**Keywords:** Markerless dynamic tumour-tracking, Intrafractional variation, Diaphragm, Lung cancer, Pancreatic cancer

## Abstract

**Background and Purpose**: For thoracic and upper abdominal tumours, respiratory motion management is essential; dynamic tumour-tracking radiotherapy (DTT-RT) is one such approach, but it typically requires fiducial marker (FM) insertion. We evaluated intra-respiratory variation in the tumour–diaphragm geometric association and its potential as a surrogate for DTT-RT.

**Materials and Methods**: Data from 22 patients (11 lung cancer [LC]; 11 locally advanced pancreatic cancer [LAPC]), treated with FM-based DTT-RT, were analysed. Ten-phase, four-dimensional computed tomography was used to quantify the 3D gross tumour volume (GTV)–FM displacement, 3D GTV–diaphragm displacement, and 3D GTV centroid displacement under free-breathing (FB). Phase-wise relative displacements for GTV–FM, GTV–diaphragm, and the GTV centroid under FB were calculated along the left–right (LR), anterior–posterior (AP), and superior–inferior (SI) axes as differences from the end-expiratory phase.

**Results**: In LC, the mean 3D GTV–FM displacement, 3D GTV–diaphragm displacement, and 3D GTV centroid displacement under FB across all phases were 1.0 (range, 0–6.9), 4.6 (0.1–19.4), and 5.8 (0.1–31.8) mm, respectively (*p* < 0.001). In LAPC, corresponding values were 1.6 (0.2–4.7), 4.5 (0.3–21.8), and 3.2 (0.4–17.0) mm (p < 0.001). Phase-wise relative GTV–diaphragm displacement was largest in the SI direction in LC and in the AP direction in LAPC, with patterns primarily driven by inspiratory-phase variations.

**Conclusions**: As a surrogate, the diaphragm exhibited larger intra-respiratory variation than FMs, primarily driven by inspiratory displacements. Incorporating phase-dependent tumour–diaphragm geometry may support markerless, diaphragm-based DTT for LC and LAPC.

## Highlights

• Tumour–diaphragm distance varied more than tumour–fiducial marker geometry.
• Increased variation was primarily driven by inspiratory phase motion.
• Displacement patterns depended on phase, axis, tumour–surrogate distance.
• The diaphragm may enable markerless dynamic tumour-tracking as a surrogate.

## Graphical abstract

**Figure:**
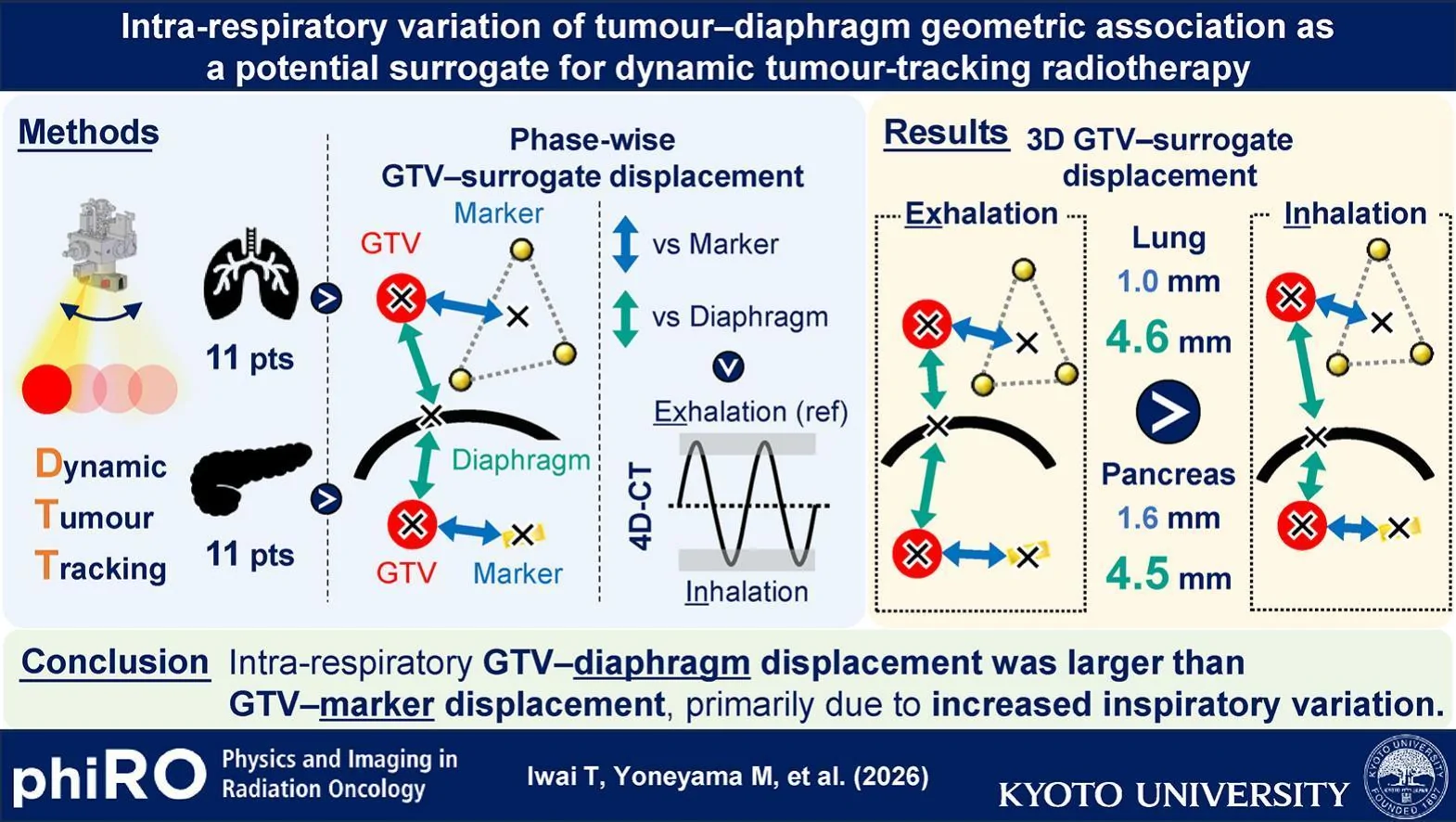

## 1 Introduction

Respiratory motion complicates accurate tumour localisation during radiotherapy for thoracic and upper abdominal tumours, including lung cancer (LC) and locally advanced pancreatic cancer (LAPC). Motion introduces discrepancies between planned and delivered dose distributions and necessitates larger target volumes [1]. To mitigate these effects and reduce radiation exposure to surrounding normal tissues, various respiratory motion management strategies, such as breath-holding, respiratory gating, abdominal compression, and dynamic tumour-tracking (DTT), have been utilized. DTT radiotherapy (DTT-RT) is considered an ideal strategy, as it allows free-breathing (FB) while enabling smaller target volumes [1], [2], [3].

DTT-RT commonly relies on invasive implantation of fiducial markers (FMs) placed in or near the tumour to monitor motion in thoracic and upper abdominal tumours [1], [3], [4], [5]. Invasive placement exposes patients to risks, such as pneumothorax, haemorrhage, and FM migration [6], [7], [8]. Moreover, many patients undergoing radiotherapy are older adults or present with limited physiological reserve, reduced pulmonary function that increases bronchoscopy risk, or impaired liver function with bleeding susceptibility [9], [10]. These considerations support avoidance of invasive procedures and highlight the need for non-invasive alternatives to FM-based DTT-RT.

Diaphragm motion serves as a promising, non-invasive surrogate for dynamic tumour motion [4], [11], [12], [13]. This strategy supports markerless tumour tracking and avoids the risks and invasiveness associated with fiducial implantation. However, current three-dimensional (3D) algorithms for estimating tumour position for DTT rely on the assumption that the geometric relationship between the FM and tumour remains constant throughout the respiratory cycle [5], [14], [15]. Nevertheless, Ueki et al. reported that intrafractional variations in the relative tumour–FM position increased toward the inspiratory phase and were associated with tumour motion amplitude and tumour–FM distance [5]. Although this uncertainty has been managed by expanding the target volume margins, larger margins increase the irradiated volume and may partially offset the dosimetric advantages of DTT in reducing normal tissue irradiation [16], [17]. These findings underscore the need to evaluate whether the tumour–diaphragm geometric relationship remains stable throughout the respiratory cycle when diaphragm-based DTT is used. Several studies have investigated diaphragm-based tumour position prediction in lung and liver cancers using kV or cone-beam computed tomography (CT) imaging [4], [18], [19], but evidence regarding intra-respiratory tumour–diaphragm geometric relationships remains limited, particularly in pancreatic cancer. Therefore, clinical application of diaphragm motion as a reliable, markerless surrogate requires assessment of the phase-dependent geometric relationship between the diaphragm and tumour.

In this study, we examined intra-respiratory variation in the geometric relationship between the moving tumour and the diaphragm using four-dimensional CT (4D-CT) in patients with LC or LAPC who underwent FM-based DTT. Furthermore, we evaluated the potential of the diaphragm to function as a surrogate for tumour respiratory motion.

## 2 Materials and methods

### 2.1 Ethical considerations and informed consent

This retrospective study was approved by the Ethics Committee of Kyoto University Graduate School and Faculty of Medicine (Approval number: R1446–2). The requirement for informed consent was waived by the opt-out method due to the retrospective study design. All procedures were performed in accordance with the ethical standards of the Ethics Committee and the Declaration of Helsinki (1975, as revised in 2013).

### 2.2 Study design and data collection

Eligibility criteria were as follows: consecutive patients with primary or metastatic LC who initiated treatment between January 2018 and September 2021 or LAPC who initiated treatment between November 2016 and March 2020; treated with stereotactic body radiation therapy with DTT using either 3D conformal radiotherapy (DTT-3D-CRT) or intensity-modulated radiotherapy (DTT-IMRT). Moreover, eligibility required FM implantation and availability of 4D-CT images. FMs included spherical gold markers (Disposable Gold Marker; Olympus Medical Systems, Tokyo, Japan) placed bronchoscopically in patients with LC, and a linear gold marker (VISICOIL, IBA dosimetry, Louvain-la-neuve, Belgium) placed endoscopically in patients with LAPC. Patients with motion-blurring artifacts at the diaphragm apex on 4D-CT images were excluded. Based on the eligibility criteria, 30 consecutive patients were assessed, of whom eight did not meet the requirements. The final analysis included a total of 22 patients: 11 with primary or metastatic LC and 11 with LAPC. Detailed patient characteristics are presented in Table 1.

**Table 1:** Patient characteristics (*N* = 22). ***Abbreviations:*** F, female; FM, fiducial marker; GTV, gross tumour volume; LAPC, locally advanced pancreatic cancer; LC, lung cancer; M, male.

Patient Number	Age (years)	Sex	Tumour location	GTV (cm^3^)	End-expiratory distance (mm)	
					GTV–FM	GTV–diaphragm
**LC**						
1	72	F	Right lower lobe	0.5	37.1	37.5
2	73	F	Right lower lobe	22.9	35.6	54.0
3	74	F	Right lower lobe	2.0	32.1	80.6
4	86	F	Right lower lobe	5.5	5.7	65.3
5	68	F	Left lower lobe	1.2	8.1	77.1
6	76	M	Right lower lobe	40.0	12.4	83.1
7	73	F	Right middle lobe	3.0	32.7	51.0
8	73	M	Left lower lobe	39.5	29.5	18.1
9	76	M	Left lower lobe	1.2	5.6	61.4
10	52	F	Right lower lobe	1.5	19.3	104.1
11	79	F	Right lower lobe	1.3	34.7	66.3
Median	73			2.0	29.5	65.3
Range	52–86			0.5–40.0	5.6–37.1	18.1–104.1
**LAPC**						
12	53	F	Pancreatic head (N0)	17.0	14.0	145.8
13	77	M	Pancreatic head (N1)	132.8	26.0	116.7
14	69	F	Pancreatic head (N0)	7.5	16.8	125.3
15	62	F	Pancreatic tail (N0)	31.1	34.3	122.4
16	69	F	Pancreatic head (N1)	23.3	38.0	127.2
17	73	M	Pancreatic head (N0)	56.8	19.5	170.4
18	72	M	Pancreatic head (N0)	24.8	40.6	163.8
19	63	M	Pancreatic tail (N1)	45.1	12.2	148.0
20	78	F	Pancreatic body (N0)	17.0	16.9	134.6
21	57	F	Pancreatic head (N0)	44.3	19.2	163.4
22	76	M	Pancreatic head (N1)	20.1	18.2	142.9
Median	69			24.8	19.2	142.9
Range	53–78			7.5–132.8	12.2–40.6	116.7–170.4

Ten-phase 4D-CT was acquired under FB conditions with 0% representing end-inspiration and 50% representing phase-defined end-expiration. Scans were acquired with a slice thickness of 2.0 or 2.5 mm and were performed with a LightSpeed RT 16 system (GE Healthcare, Chicago, USA) until July 2017, and with a SOMATOM Definition AS CT simulator (Siemens Healthineers, Erlangen, Germany) thereafter. The 50% end-expiratory phase was used as the reference phase for all analyses. A real-time positioning management system (Varian Medical Systems, Palo Alto, California, USA) was used to monitor each patient's breathing pattern. For patients with LAPC, contrast agents were administered during CT acquisition.

Gross tumour volumes (GTVs) were based primarily on clinical treatment plans. For respiratory phases of the 4D-CT images not used in treatment planning, GTVs were delineated by one of two board-certified radiation oncologists for each case, covering all ten phases (0–90%). Inter-observer variability was not quantitatively assessed. For LC, the GTV included primary or metastatic solitary pulmonary tumours. For LAPC, both the primary tumour and metastatic lymph nodes were delineated. Additionally, the ipsilateral diaphragm apex in LC and right diaphragm in LAPC were automatically segmented on all 4D-CT images. Segmentation was performed 1 cm caudal to the diaphragm apex, using a threshold of −150 to 200 Hounsfield Units, with the volume of interest (VOI) function in Eclipse™ (version 16.0, Varian Medical Systems, Palo Alto, California, USA) (**Supplementary Fig. 1**).

### 2.3 3D GTV–FM displacement, GTV–diaphragm displacement, and GTV centroid displacement under FB

**Fig. 1:**
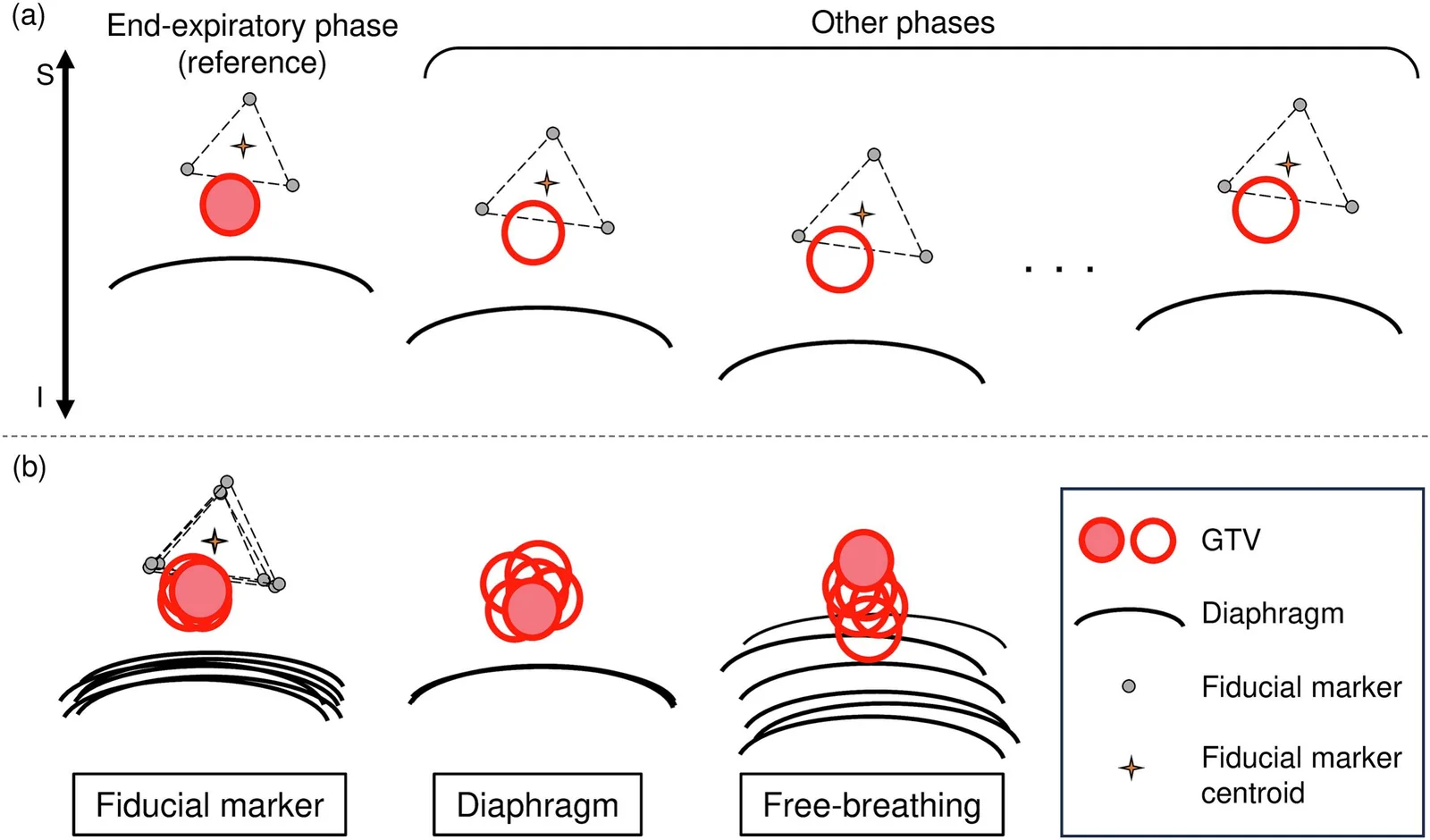
Schematic illustration of (a) the geometric associations among the moving tumour, FM, and diaphragm across the respiratory cycle; and (b) GTV–FM displacement, GTV–diaphragm displacement, and GTV centroid displacement under FB. ***Abbreviations:*** GTV, gross tumour volume; FB, free-breathing; FM, fiducial marker; I, inferior; S, superior.

To evaluate the association between dynamic tumour positioning and its surrogates across ten respiratory phases, we analysed 3D displacements (i) between the GTV centroid and the FM centroid and (ii) between the GTV centroid and the diaphragm apex at each respiratory phase (Fig. 1). These measurements were referred to as 3D GTV–FM displacement and 3D GTV–diaphragm displacement, respectively. The diaphragm apex was defined as the centroid of the most cranial slice, chosen as a reproducible radiographic landmark that reflects local diaphragm motion while being less affected by deformation of the overall diaphragm contour. Furthermore, for comparison, the 3D GTV centroid displacement under FB was assessed using the original 4D-CT dataset without positional adjustment.

To calculate 3D GTV–FM displacement, rigid image registration was performed using the centroid of multiple spherical gold markers in LC cases and the midpoint of a linear gold marker in LAPC cases, reflecting the clinical standard for DTT treatment planning [20], [21], [22]. Regarding 3D GTV–diaphragm displacement, rigid image registration was performed along left–right (LR), anterior–posterior (AP), and superior–inferior (SI) axes across all ten respiratory phases of the 4D-CT images, using the automatically segmented diaphragm apex in Eclipse™.

Phase-wise relative displacements for the GTV–FM, GTV–diaphragm, and the GTV centroid under FB were calculated along the LR, AP, and SI axes as differences from the 50% end-expiratory phase, which was defined as zero. For phase-wise relative GTV–FM and GTV–diaphragm displacements, negative and positive values indicated displacement toward and away from the corresponding surrogate, respectively, and the numbers of observations outside ±2 and ± 5 mm in each direction were counted. For the phase-wise relative GTV centroid displacement under FB, toward patient right, anterior, and inferior displacements were defined as negative, and toward patient left, posterior, and superior displacements were defined as positive. Expiratory motion covered 30–70% of the respiratory cycle, with the remaining phases classified as inspiratory.

### 2.4 Statistical analysis

The Friedman test was used to compare the 3D GTV–FM displacement, 3D GTV–diaphragm displacement, and 3D GTV centroid displacement under FB. When the Friedman test indicated a difference, post-hoc pairwise comparisons were performed using the Wilcoxon signed-rank test with Bonferroni correction. For each comparison family, statistical thresholds were based on a family-wise alpha level of 0.05, and adjusted *p* values were reported.

A linear mixed-effects model was applied to evaluate whether phase-wise relative GTV–diaphragm displacement patterns differed according to motion axis, tumour type, and the end-expiratory GTV–diaphragm distance group. Phase-wise relative displacement was treated as the dependent variable. The respiratory phase, axis (LR, AP, or SI), tumour type (LC or LAPC), and end-expiratory GTV–diaphragm distance group were included as fixed effects. For this exploratory analysis, the distance group was dichotomized at the cohort-specific median to simplify interpretation of distance-dependent displacement patterns and to reduce the influence of extreme values in this small cohort. Interaction terms were examined to determine whether displacement patterns varied by tumour type, axis, or distance group.

All statistical analyses were performed using R software (version 4.5.2, R Foundation for Statistical Computing, Vienna, Austria).

## 3 Results

In the LC cohort (*N* = 11), the mean 3D GTV–FM displacement, 3D GTV–diaphragm displacement, and 3D GTV centroid displacement under FB across all respiratory phases were 1.0 ± 1.2 (range, 0.0–6.9; 95th percentile, 3.0), 4.6 ± 4.5 (0.1–19.4; 14.1), and 5.8 ± 6.8 (0.1–31.8; 18.2) mm, respectively. The 3D GTV–FM displacements were significantly smaller than the other two 3D displacements (*p* < 0.001 for both comparisons). The 3D GTV–diaphragm displacements were also significantly smaller than the 3D GTV centroid displacement under FB (*p* = 0.004).

In the LAPC cohort (*N* = 11), the mean 3D GTV–FM displacement, 3D GTV–diaphragm displacement, and 3D GTV centroid displacement under FB across all respiratory phases were 1.6 ± 0.9 (range, 0.2–4.7; 95th percentile, 3.3), 4.5 ± 4.6 (0.3–21.8; 13.3), and 3.2 ± 3.1 (0.4–17.0; 8.6) mm, respectively. The 3D GTV–FM displacements were significantly smaller than the other two 3D displacements (*p* < 0.001 for both comparisons), whereas the 3D GTV centroid displacements under FB were significantly smaller than the 3D GTV–diaphragm displacements (*p* = 0.03).

**Fig. 2:**
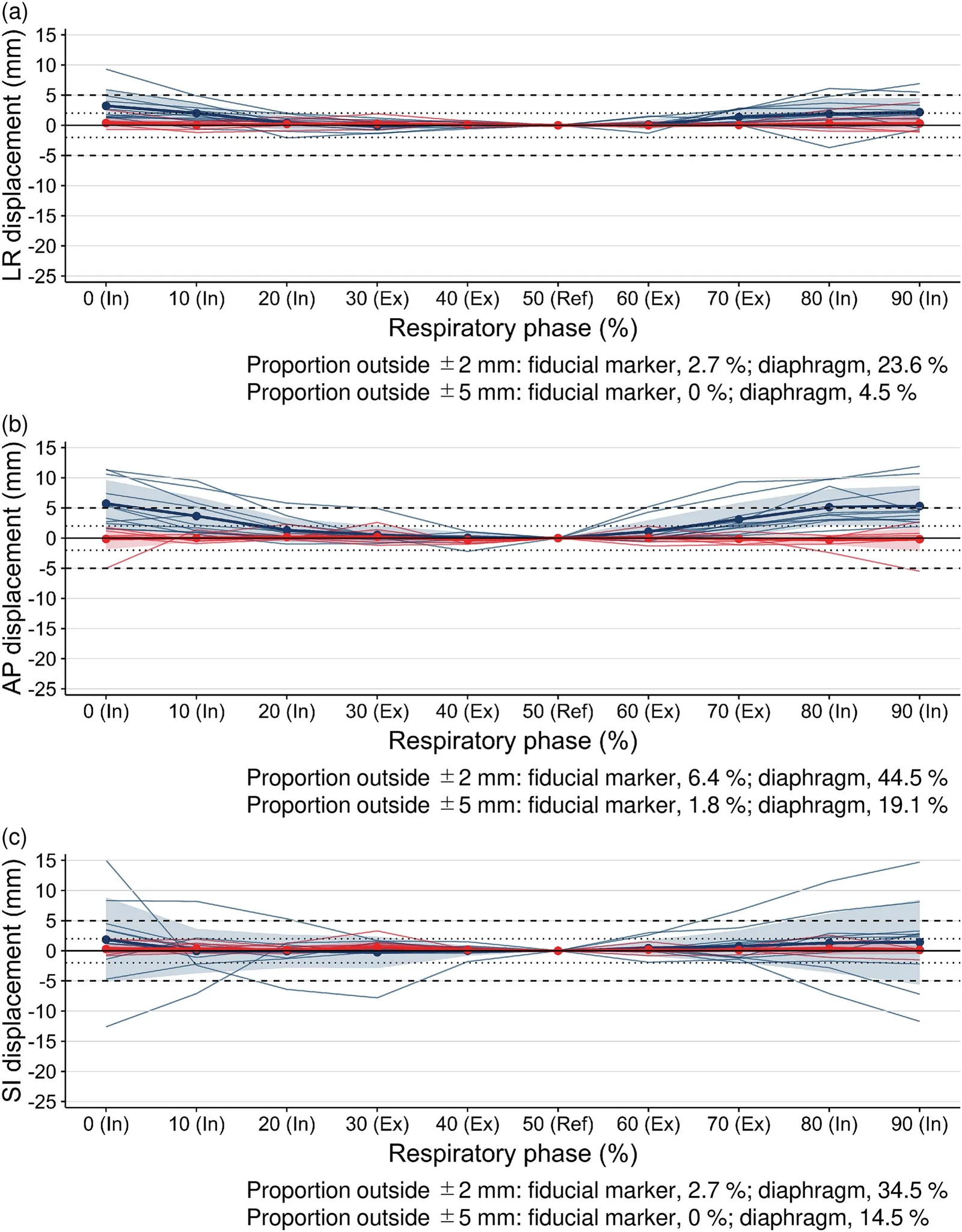
Spaghetti plots of phase-wise relative GTV–FM and GTV–diaphragm displacements in patients with LC, referenced to the end-expiratory phase (50%) in the (a) LR, (b) AP, and (c) SI directions. ***Notes:*** Each line represents an individual patient (red, GTV–FM; blue, GTV–diaphragm). Bold lines indicate the cohort mean at each respiratory phase, with shaded areas representing the mean ± standard deviation (SD). Negative and positive values indicate displacement toward and away from the corresponding surrogate, respectively. Black dashed and dotted lines indicate ±5 mm and ± 2 mm, respectively. ***Abbreviations:*** AP, anterior–posterior; Ex, expiratory phase; In, inspiratory phase; LR, left–right; LC, lung cancer; Ref, reference; SI, superior–inferior. The other abbreviations are the same as in Fig. 1. (For interpretation of the references to colour in this figure legend, the reader is referred to the web version of this article.)

**Fig. 3:**
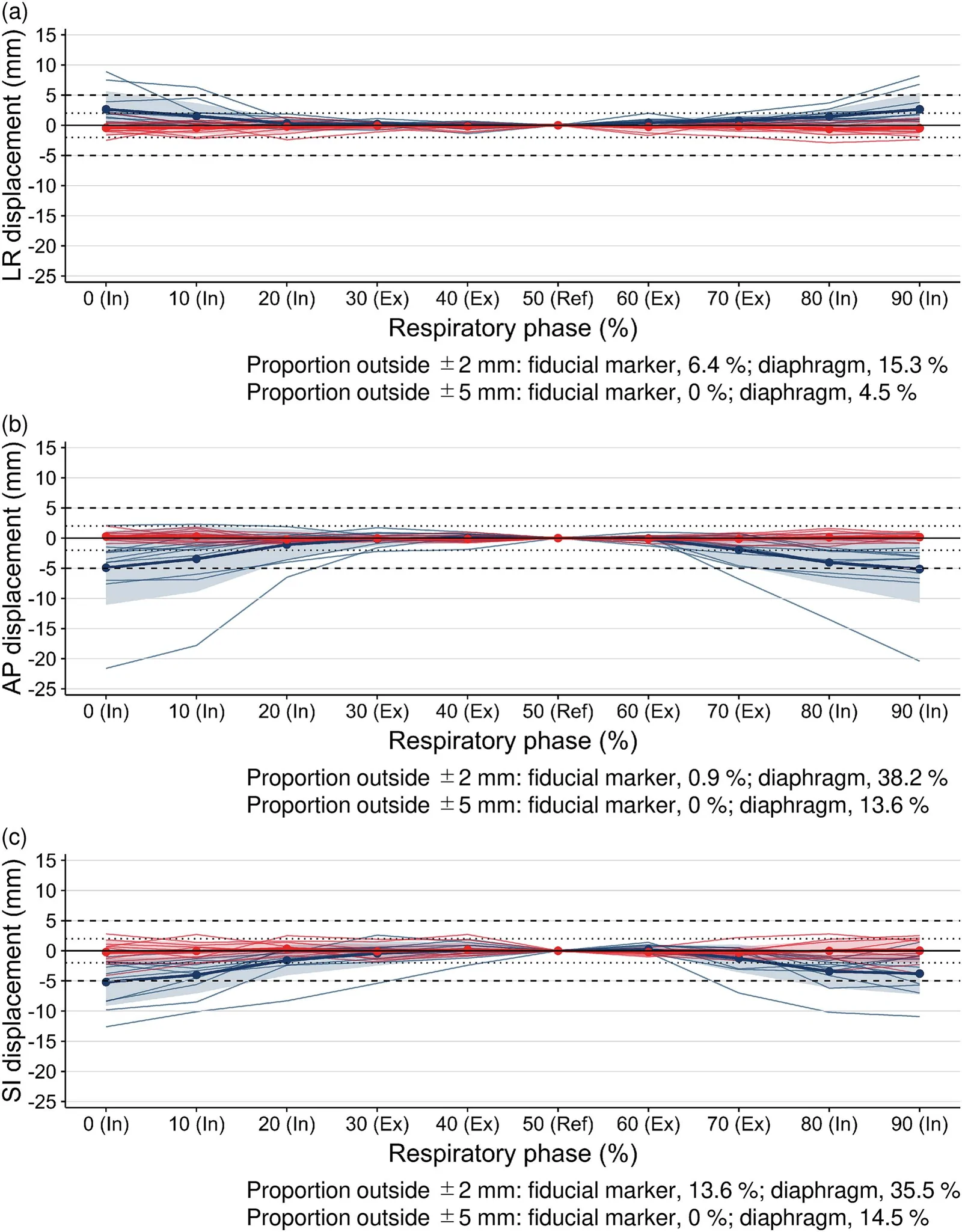
Spaghetti plots of phase-wise relative GTV–FM and GTV–diaphragm displacements in patients with LAPC, referenced to the end-expiratory phase (50%) in the (a) LR, (b) AP, and (c) SI directions. **Notes** are the same as in Fig. 2. ***Abbreviations:*** LAPC, locally advanced pancreatic cancer. The other abbreviations are the same as in Fig. 1, Fig. 2.

Table 2 summarizes the phase-wise relative displacements in LC and LAPC. Additionally, Fig. 2, Fig. 3 show the patient-level phase-wise relative GTV–FM and GTV–diaphragm displacements, along with the overall percentage of observations outside ±2 mm and ± 5 mm in LC and LAPC. In LC, the range of phase-wise relative GTV–diaphragm displacements was widest in the SI direction, followed by the AP and LR directions. All cases showed greater inspiratory than expiratory displacement in the LR and AP directions. Conversely, SI-directional patterns were heterogeneous: eight of 11 cases exhibited larger inspiratory and smaller expiratory displacement, while the remaining three cases showed the opposite trend.

**Table 2:** Descriptive statistics of phase-wise relative GTV–FM displacement, GTV–diaphragm displacement, and GTV centroid displacement under FB. ***Notes:*** For phase-wise relative GTV–FM and GTV–diaphragm displacements, negative and positive values indicated displacement toward and away from the corresponding surrogate, respectively. For the phase-wise relative GTV centroid displacement under FB, toward patient right, anterior, and inferior displacements were defined as negative, and toward patient left, posterior, and superior displacements were defined as positive. ***Abbreviations:*** AP, anterior–posterior; FB, free-breathing; LR, left–right; SD, standard deviation; SI, superior–inferior. The other abbreviations are the same as in Table 1.

**Phase-wise relative displacement** (mm)	Direction	Mean ± SD	Range	5th–95th percentile
**LC**				
**GTV–FM displacement**	LR	0.2 ± 0.7	−1.2–3.8	−0.7–1.2
	AP	−0.1 ± 1.1	−5.5–2.7	−1.1–1.4
	SI	0.3 ± 0.7	−1.5–3.3	−0.7–1.6
**GTV–diaphragm displacement**	LR	1.2 ± 2.0	−3.7–9.3	−1.0–5.0
	AP	2.9 ± 3.3	−2.2–11.9	−0.2–9.9
	SI	0.6 ± 4.1	−12.6–15.0	−7.1–8.1
**GTV centroid displacement under FB**	LR	0.1 ± 1.3	−5.4–3.5	−2.7–1.6
	AP	−0.9 ± 1.5	−6.3–2.0	−4.0–1.3
	SI	−5.3 ± 6.9	−31.5–1.7	−17.6–0.7
**LAPC**				
**GTV–FM displacement**	LR	−0.3 ± 0.9	−2.9–2.1	−2.0–0.8
	AP	0.0 ± 0.7	−1.4–2.0	−0.9–1.2
	SI	0.0 ± 1.4	−4.1–2.8	−2.1–2.5
**GTV–diaphragm displacement**	LR	1.1 ± 1.8	−1.4–8.9	−0.6–4.7
	AP	−2.3 ± 4.1	−21.6–2.3	−7.4–1.0
	SI	−2.1 ± 3.1	−12.6–2.6	−8.6–1.0
**GTV centroid displacement under FB**	LR	0.5 ± 0.9	−1.2–3.4	−0.6–2.3
	AP	−1.0 ± 1.2	−5.2–0.8	−2.9–0.5
	SI	−2.6 ± 3.1	−16.4–1.1	−8.0–0.3

In LAPC, the range of phase-wise relative GTV–diaphragm displacements was widest in the AP direction, followed by the SI and LR directions. Nine of 11 cases demonstrated greater inspiratory than expiratory displacement in the LR direction, while the AP and SI showed smaller displacement during inspiration than expiration in 10 and all cases, respectively.

**Fig. 4:**
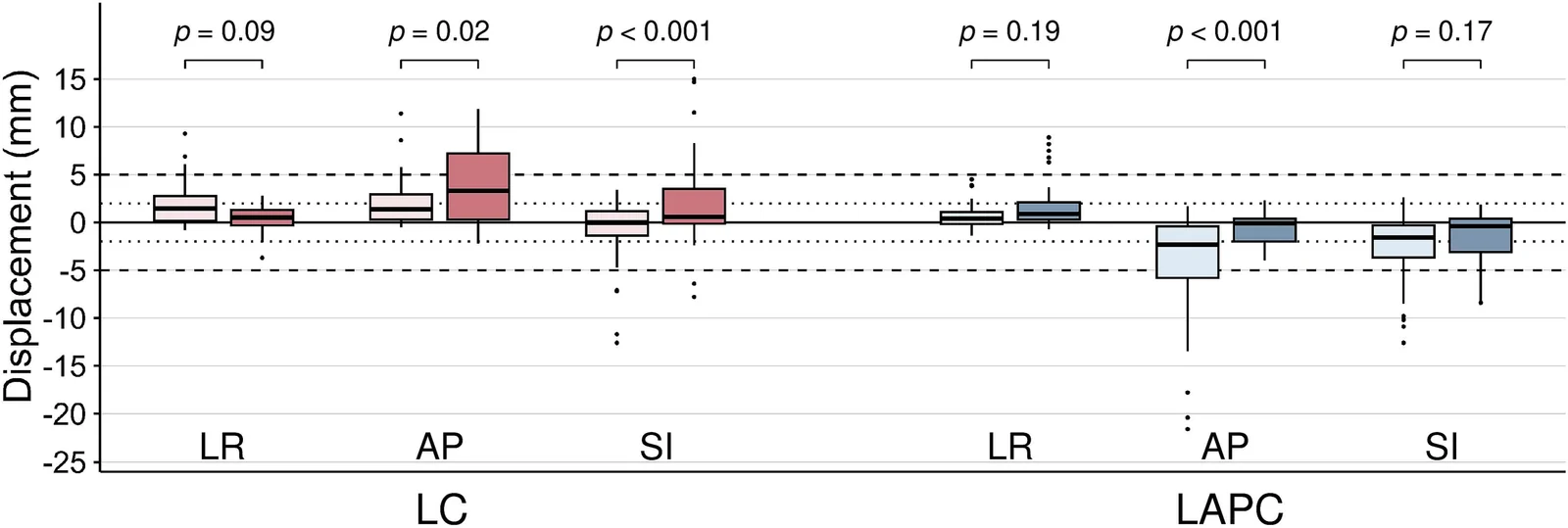
Boxplots of phase-wise relative GTV–diaphragm displacement, referenced to the end-expiratory phase (50%) as zero in the (a) LR, (b) AP, and (c) SI directions. ***Notes:*** Colours indicate groups above or below the median end-expiratory GTV–diaphragm distance in each cohort: 65.3 mm for LC and 142.9 mm for LAPC. Red/light red indicate above/below the median in LC, and dark blue/light blue indicate above/below the median in LAPC, respectively. ***Abbreviations:*** AP, anterior–posterior; GTV, gross tumour volume; LR, left–right; LAPC, locally advanced pancreatic cancer; LC, lung cancer; SI, superior–inferior. (For interpretation of the references to colour in this figure legend, the reader is referred to the web version of this article.)

A linear mixed-effects model identified a significant interaction among the end-expiratory GTV–diaphragm distance group, motion axis, and tumour type (*p* = 0.002), indicating that end-expiratory GTV–diaphragm distance-dependent displacement patterns differed between the LC and LAPC cohorts. In the LC cohort, significant GTV–diaphragm distance-dependent differences were observed in the AP and SI directions (*p* = 0.02 and *p* < 0.001, respectively), with the difference being more pronounced in the SI direction, whereas no significant difference was observed in the LR direction (*p* = 0.09). By contrast, in the LAPC cohort, significant GTV–diaphragm distance-dependent differences were confined to the AP direction (p < 0.001), with no marked differences in the LR or SI directions (*p* = 0.19 and 0.17, respectively). Boxplots of the phase-wise relative GTV–diaphragm displacements are shown in Fig. 4.

## 4 Discussion

This study demonstrated that intra-respiratory geometric variation between the tumour and diaphragm was greater than that between the tumour and FMs, and that this variation was also influenced by the GTV–diaphragm distance and tumour site, such as lung or pancreatic tumours. These findings suggest that correction for tumour–diaphragm geometric variation may be necessary when using the diaphragm as a surrogate for DTT-RT.

Current DTT strategies use a surrogate under the assumption that its motion is synchronous with the tumour's respiratory motion. FMs, implanted near the tumour, are expected to maintain a strong geometric association with tumour motion. Previous research has found minimal residual tumour motion relative to the FMs from end-expiration, with mean values of 0.1, 0.3, and 0.0 mm in the LR, AP, and SI directions, indicating high tumour–FM synchrony [5]. Our findings are consistent with these previous data. By contrast, our results showed that when the diaphragm is used as the surrogate, the tumour–surrogate distance increases during inspiration compared with expiration, indicating that perfect synchrony does not hold. Existing 3D tumour position estimation algorithms for DTT assume that the surrogate centroid moves synchronously with the tumour; when asynchrony occurs, margin expansion is required for compensation. Such expansion can increase the normal-tissue dose and risk of treatment-related toxicity [23], [24]. Because phase-dependent variation in the tumour–diaphragm relationship has not been fully characterized, an estimation framework that explicitly accounts for both inspiratory and expiratory phases is needed. These findings suggest that incorporating a variable offset vector into DTT may be useful for handling phase-dependent diaphragm–tumour asynchrony. In our previous study, the 90th percentile tumour-position prediction errors obtained using respiratory signals and variable offset vectors were within 1 mm of those obtained assuming direct tumour identification in all directions, supporting the potential clinical utility of this approach [25].

Diaphragm-based, markerless DTT has been investigated mainly in lung and liver [4], [18], [19], whereas its suitability for pancreatic cancer remains uncertain, because pancreatic tumours show complex, multidirectional respiratory motion [26], [27]. Previous reports have shown that the diaphragm reflects pancreatic SI motion well, suggesting its potential use as an SI surrogate [28]. However, pancreatic motion can also be substantial in the AP and LR directions. Minn et al. have shown that single-scan 4D-CT ranges reached 28.8, 13.7, and 7.6 mm in the SI, LR, and AP directions, respectively [29]; the corresponding ranges in our study were 17.5, 4.6, and 6.0 mm, respectively. They also reported that maximum intrafractional displacements were 48.8, 41.3, and 68.1 mm in the SI, LR, and AP directions, respectively, which exceed typical LR and AP amplitudes reported for lung or liver tumours.

In LAPC, phase-wise relative GTV–diaphragm displacement patterns in the LR and SI directions were relatively consistent regardless of the end-expiratory GTV–diaphragm distance, whereas shorter distances were associated with greater negative inspiratory displacements in the AP direction. In LC, these patterns differed by the end-expiratory GTV–diaphragm distance group, with greater inspiratory positive displacements in patients with longer distances, particularly in the SI and AP directions. Additionally, in LAPC, phase-wise relative GTV–diaphragm displacement showed a wider range than the GTV centroid displacement under FB, particularly in the AP direction, suggesting substantial AP asynchrony between diaphragm and tumour motion. This may explain why the diaphragm-tracking scenario showed larger errors than the FB scenario. These findings suggest that diaphragm-based markerless DTT could be feasible for LAPC with appropriate phase-dependent offset correction.

Because our analysis was based on a single 4D-CT acquisition per patient, intra- and inter-fractional motion could not be fully assessed. Moreover, abdominal organs are subject to substantial inter-fractional variation due to changes in adjacent bowel [30]. Accordingly, the observed displacements should be interpreted as representing phase-dependent geometric variation within a single 4D-CT session rather than as comprehensive estimates of motion variability throughout treatment. Residual errors approaching 5 mm would be clinically relevant in stereotactic body radiotherapy and should not be regarded as equivalent to FM-based tracking because such errors may require additional margins or wider tracking tolerances. For clinical implementation, the tumour–diaphragm offset vector should not be treated as a fixed parameter. Instead, real-time or fraction-specific recalibration would be required to account for intra- and inter-fractional anatomical changes. Such recalibration could be incorporated into adaptive radiotherapy workflows, in which the offset vector is generated and updated for each fraction.

This study has several limitations. First, our analyses relied on a single 4D-CT acquisition per patient. Intrafraction motion during treatment can exceed and is not fully predicted by 4D-CT [29]; thus, a single acquisition may not capture intra- and inter-fractional variations. Second, the small sample size limits the robustness of the data and reliability of findings regarding the utility of the variable offset vector. Therefore, the proposed patient-specific phase-dependent offset correction remains preliminary and requires further validation in larger cohorts. Moreover, most lesions in our LAPC cohort were located in the pancreatic head, and motion heterogeneity across pancreatic subsites is well recognized [31]. These findings should not be extrapolated to pancreatic body/tail tumours; further validation in these subsites is needed. Third, excluding patients with motion-blurred images may have introduced selection bias by underrepresenting patients with irregular breathing, prolonged respiratory cycles, or deep inspiration. Consequently, our findings may predominantly reflect ideal candidates with regular, well-defined diaphragm motion. Fourth, observer variability may have influenced results, as GTVs were contoured by one of two board-certified radiation oncologists for each case, whereas the diaphragm apex was automatically segmented using a VOI, and image registration was performed using standardized procedures. Inter-observer variability was not quantitatively assessed. Additionally, SI localization errors may have been affected by slice thickness, potentially affecting GTV delineation and image registration. However, the impact on registration was considered limited as the diaphragm registration region included multiple voxels over a 1-cm SI extent.

In conclusion, intra-respiratory GTV–diaphragm displacements exceeded GTV–FM displacements, driven mainly by inspiratory increases in tumour–diaphragm displacements. Because the dominant motion axes differed between the LC and LAPC cohorts and displacement patterns depended on the end-expiratory GTV–diaphragm distance, patient-specific phase-dependent offset correction along all three axes may be needed for diaphragm-based DTT. In practice, such correction could be derived from the planning 4D-CT or cone-beam CT by estimating phase-specific tumour–diaphragm offset vectors and incorporating them into markerless tumour position prediction. With further development and validation, diaphragm-based markerless DTT may be feasible for selected patients with LC and LAPC.

## Data availability statement for this work

The data are not publicly available due to privacy or ethical restrictions.

## Declaration of generative AI use

The authors used ChatGPT (OpenAI) for language translation and for generation and modification of analysis scripts. After using this tool, the authors reviewed and edited the content as needed and take full responsibility for the content of the publication.

## CRediT authorship contribution statement

**Takahiro Iwai:** Writing – review & editing, Writing – original draft, Visualization, Validation, Methodology, Investigation, Formal analysis, Data curation, Conceptualization. **Masahiro Yoneyama:** Writing – review & editing, Writing – original draft, Visualization, Validation, Methodology, Investigation, Formal analysis, Data curation, Conceptualization. **Noriko Kishi:** Writing – review & editing, Validation, Supervision, Resources, Project administration, Methodology, Conceptualization. **Takanori Adachi:** Writing – review & editing, Supervision, Investigation, Data curation. **Mitsuhiro Nakamura:** Writing – review & editing, Validation, Project administration, Methodology, Conceptualization. **Yukine Shimizu:** Writing – review & editing, Conceptualization. **Hideaki Hirashima:** Writing – review & editing, Data curation. **Nobutaka Mukumoto:** Writing – review & editing, Conceptualization. **Michio Yoshimura:** Writing – review & editing, Resources. **Takashi Mizowaki:** Writing – review & editing, Funding acquisition.

## Funding sources

This work was supported by the 10.13039/100009619Japan Agency for Medical Research and Development (AMED) [Grant Number JP24ck0106924].

## Declaration of competing interest

The authors declare the following financial interests/personal relationships which may be considered as potential competing interests: **Takahiro Iwai:** Employment under a joint research agreement funded by Varian Medical Systems; **Noriko Kishi**: Honoraria for lecture from Varian Medical Systems, and employment under a joint research agreement funded by Hitachi High-Tech Co., Ltd.; **Mitsuhiro Nakamura**: Research grants and scholarship donations from Hitachi High-Tech Co., Ltd.; inventor on issued and pending patents related to markerless tumour tracking; **Takashi Mizowaki:** Research grants and scholarship donations from Hitachi High-Tech Co., Ltd.; commissioned and collaborative research projects and honoraria with Varian Medical Systems; commissioned projects and honoraria with Brainlab AG; and honoraria from Elekta and Hitachi Ltd.

## Appendix A Supplementary data

Supplementary material 1 **Supplementary Figure 1**. Example of the diaphragm-apex structure VOI: (a) coronal and (b) sagittal images. ***Notes:*** The red shading denotes the diaphragm-apex VOI. The two white dashed lines indicate the apex level and a plane 1 cm caudal to it. ***Abbreviations:*** A, anterior; L, left; P, posterior; R, right; VOI, volume of interest.

Supplementary Video 1 **Supplementary Video 1.** 3D plots of mean phase-wise GTV–diaphragm displacement, referenced to the end-expiratory phase (50%) as zero, shown as a rotating three-dimensional visualisation. ***Notes:*** Colours indicate groups above or below the median end-expiratory GTV–diaphragm distance in each cohort: 65.3 mm for LC and 142.9 mm for LAPC. Red/light red indicate above/below the median in LC, and dark blue/light blue indicate above/below the median in LAPC, respectively. Shaded areas represent the 5th–95th percentile range. ***Abbreviations:*** 3D, three-dimensional. The other abbreviations are the same as in Figures 1–3.
