# Supplementary material for: Intra-respiratory variation in tumour–diaphragm geometric association as a potential surrogate for dynamic tumour-tracking radiotherapy
Source: Phys Imaging Radiat Oncol. 2026 Aug 4;40:101057. doi: 10.1016/j.phro.2026.101057 (PMC13551932; doi:10.1016/j.phro.2026.101057)
Supplement: Supplementary file 1 [file mmc1.pdf]

Supplementary files

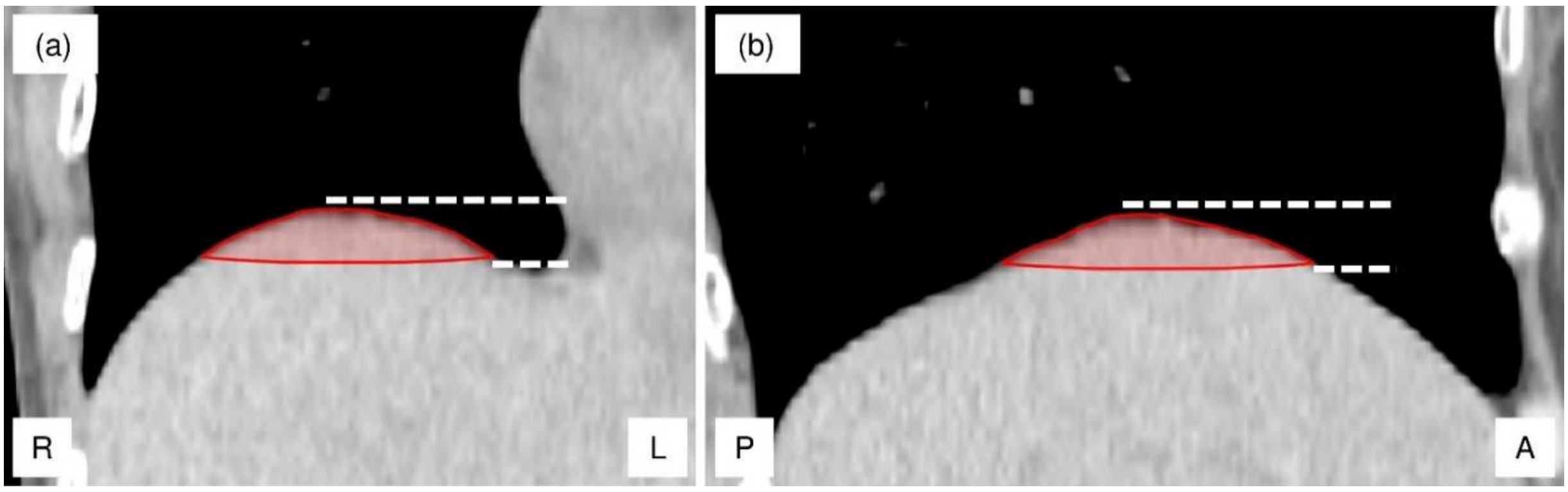

Supplementary Figure 1. Example of the diaphragm-apex structure VOI: (a) coronal and (b) sagittal images

Notes: The red shading denotes the diaphragm-apex VOI. The two white dashed lines indicate the apex level and a plane 1 cm caudal to it.

Abbreviations: A, anterior; L, left; P, posterior; R, right; VOI, volume of interest.

Supplementary Video 1. 3D plots of mean phase-wise GTV–diaphragm displacement, referenced to the end-expiratory phase (50%) as zero, shown as a rotating three-dimensional visualisation.

Notes: Colours indicate groups above or below the median end-expiratory GTV–diaphragm distance in each cohort: 65.3 mm for LC and 142.9 mm for LAPC. Red/light red indicate above/below the median in LC, and dark blue/light blue indicate above/below the median in LAPC, respectively. Shaded areas represent the 5th–95th percentile range.

Abbreviations**:** 3D, three-dimensional. The other abbreviations are the same as in **Figures 1–3**.
